# Geographical and Temporal Diversity of ‘*Candidatus* Phytoplasma solani' in Wine-Growing Regions in Slovenia and Austria

**DOI:** 10.3389/fpls.2022.889675

**Published:** 2022-05-20

**Authors:** Nataša Mehle, Sanda Kavčič, Sara Mermal, Sara Vidmar, Maruša Pompe Novak, Monika Riedle-Bauer, Günter Brader, Aleš Kladnik, Marina Dermastia

**Affiliations:** ^1^Department of Biotechnology and Systems Biology, National Institute of Biology, Ljubljana, Slovenia; ^2^Faculty of Viticulture and Enology, University of Nova Gorica, Vipava, Slovenia; ^3^Federal College and Research Institute for Viticulture and Pomology Klosterneuburg, Klosterneuburg, Austria; ^4^Center for Health & Bioresources, Austrian Institute of Technology, Tulln, Austria; ^5^Department of Biology, Biotechnical Faculty, University of Ljubljana, Ljubljana, Slovenia

**Keywords:** Bois noir, genotyping, ‘*Ca*. P. solani', *tuf* gene, *sec*Y, survey, tuf-b2, stamp

## Abstract

As the causal agent of the grapevine yellows disease Bois noir, ‘*Candidatus* Phytoplasma solani' has a major economic impact on grapevines. To improve the control of Bois noir, it is critical to understand the very complex epidemiological cycles that involve the multiple “*Ca*. P. solani” host plants and insect vectors, of which *Hyalesthes obsoletus* is the most important. In the present study, multiple genotyping of the *tuf*, *secY, stamp*, and *vmp1* genes was performed. This involved archived grapevine samples that were collected during an official survey of grapevine yellows throughout the wine-growing regions of Slovenia (from 2003 to 2016), plus samples from Austrian grapevines, stinging nettle, field bindweed, and insect samples (collected from 2012 to 2019). The data show that the tuf-b2 type of the *tuf* gene has been present in eastern Slovenia since at least 2003. The hypotheses that the occurrence of the haplotypes varies due to the geographical position of Slovenia on the Italian–Slovenian Karst divide and that the haplotypes are similar between Slovenian and Austrian Styria were confirmed. The data also show haplotype changes for host plants and *H*. *obsoletus* associated with ‘*Ca*. P. solani,' which might be linked to new epidemiological cycles of this phytoplasma that involve not just new plant sources and new insect vectors, but also climate and land-use changes.

## Introduction

Bois noir (BN) is an important economic disease of grapevines (*Vitis vinifera*). Its causal agent is a phytopathogenic bacterium “*Candidatus* Phytoplasma solani” (taxonomic subgroup 16SrXII-A) from the order Acholeplasmatales in the class Mollicutes (Quaglino et al., 2013). This phytoplasma is endemic to the wider Mediterranean region and the southern half of Europe (Johannesen et al., 2012; Aryan et al., 2014; Plavec et al., 2015; Trivellone et al., 2016; Balakishiyeva et al., 2018). It has also been occasionally reported from China, Chile, Canada (Dermastia et al., 2017), and other countries (“*Candidatus* Phytoplasma solani” (PHYPSO) [World distribution] | EPPO Global Database). The “*Ca*. P. solani” has a broad host range that includes more than 90 wild and host plants species cultivated from 36 families.

In western and central Europe, stinging nettle (*Urtica dioica*) and bindweed (*Convolvulus arvensis*) can host “*Ca*. P. solani”, and these plants are the reproduction hosts of the main vector, the planthopper *Hyalesthes obsoletus* Signoret (Hemiptera: Cixiidae). The nymphs of *H. obsoletus* develop underground on the roots of *U. dioica* and *C. arvensis*, where they acquire the phytoplasma and complete their latency period. Recent studies in southeastern Europe have indicated additional plant species that simultaneously harbor the phytoplasma and enable *H. obsoletus* reproduction, namely *V. agnus-castus* and *Crepis foetida*. The vectoring ability of “*Ca*. P. solani” by *H. obsoletus* from infected *V. agnus-castus* and *C. foetida* has been experimentally confirmed (Kosovac et al., 2016, 2018, 2019). Unlike the nymphs, which are restricted to this narrow host range, the adults of *H. obsoletus* are polyphagous. Therefore, they can transmit the pathogen from their nymphal hosts to a wide range of cultivated and wild plants, including grapevines. Thus, in western and central Europe, *U. dioica* and *C. arvensis* are of particular importance for “*Ca*. P. solani” epidemiology (Langer and Maixner, 2004; Aryan et al., 2014; Landi et al., 2015). Due to their restriction to a few reproduction hosts, the nymphs are not considered as able to acquire phytoplasma from crop plants. In consequence, crop species (including grapevines) are dead-end hosts in epidemic cycles with *H. obsoletus* as the pathogen vector (Johannesen et al., 2008; Jović et al., 2019).

Besides *H. obsoletus*, there are probably other Auchenorrhyncha vectors of “*Ca*. P. solani”, with transmission to grapevine reported for some, and particularly for *Reptalus* spp. (Cvrković et al., 2014; Oliveri et al., 2015; Chuche et al., 2016; Trivellone et al., 2016; Balakishiyeva et al., 2018; Mitrovic et al., 2019). The role of these species in BN transmission has not been fully elucidated.

Over the last 15 years, molecular characterization of “*Ca*. P. solani” types in wild and cultivated host plants and in the transmitting insect species has greatly increased our knowledge of BN epidemiology. Analysis of the elongation factor TU gene sequences of “*Ca*. P. solani” strains have identified two major lineages, tuf-a and tuf-b, which are associated with *U. dioica* and *C. arvensis*, respectively. Two independent epidemic cycles with these two hosts exist in the field (Langer and Maixner, 2004). Recent studies in Austria have revealed that a genealogical intermediate between the tuf-a and tuf-b types, namely tuf-b2, is associated with *U. dioica* isolates of “*Ca*. P. solani” (Aryan et al., 2014). Subsequently, the phytoplasma type tuf-b2 was also confirmed for “*Ca*. P. solani” strains from grapevines and planthoppers in Croatia, Macedonia, Montenegro, and Azerbaijan (Atanasova et al., 2015; Plavec et al., 2015; Kosovac et al., 2016; Balakishiyeva et al., 2018). Interestingly, natural infections with *U. dioica*-associated phytoplasmas (i.e., tufa, tuf-b2 types) have been reported almost exclusively from *U. dioica* and grapevines. In contrast, the *C. arvensis* types (i.e., “classical” tuf-b type; designated here as tuf-b1) and the recently described tuf-b3 (Balakishiyeva et al., 2018) infect a wide host range. In addition, other genes have been used to analyze the genetic diversity of BN types. One of these is *secY*, which encodes an important membrane unit of the secretory pathway (Fialová et al., 2009; Aryan et al., 2014; Cvrković et al., 2014; Plavec et al., 2015, 2018; Balakishiyeva et al., 2018), where genotyping also allows discrimination between *U. dioica* and *C. arvensis* types (Aryan et al., 2014). Nevertheless, *tuf* and *secY* are fewer variables than two genes that encode membrane proteins; namely, *vmp1* and *stamp* (Cimerman et al., 2009; Fabre et al., 2011). Here, the *stamp* gene is subject to positive selection pressure and might be involved in the interaction of phytoplasma with its insect vector (Fabre et al., 2011). Therefore, analysis of *vmp1* and *stamp* genes allows finer differentiation of “*Ca*. P. solani” strains, and, so far, 80 *vmp1* and 46 *stamp* gene variants have been described (Martini et al., 2019). Several reports suggest that *vmp1* and *stamp* are useful for epidemiological studies on BN (Aryan et al., 2014; Murolo et al., 2014; Oliveri et al., 2015; Plavec et al., 2015; Pierro et al., 2019).

Genetic analysis of *H. obsoletus* populations from European countries and Israel have not revealed any affiliations between the host plants and the insect mitochondrial DNA haplotypes, which implies that *H. obsoletus* can use both, *U. dioica* and *C. arvensis*, as developmental hosts. Nevertheless, subsequent analysis using random amplification of polymorphic DNA and simple sequence repeat markers in midwest Germany provided evidence for genetically divergent host populations (i.e., a host race) (Johannesen et al., 2008; Imo et al., 2013).

Overall, the importance of “*Ca*. P. solani” as a major pathogen can be attributed to several factors: the wide host range of the phytoplasma, which includes herbaceous and woody plant species; the use of some of these species by *H. obsoletus* as developmental hosts; and the polyphagy of adult *H. obsoletus*, which leads to the transmission of the phytoplasma to different crops.

The first reports of BN in western and central Europe date back to the 1960s, when BN was detected in France and the Rhine and Mosel regions of Germany. The first epidemic outbreaks in France and Germany were in the 1990s, with an epidemic outbreak in Austria around 2000; these were associated with *C. arvensis*. In contrast, vector populations associated with *U. dioica* were reported from northern Italy as early as around 2000. Then in the last 20 years, *U. dioica*-associated BN cycles have increasingly caused epidemic outbreaks in Germany, Switzerland, northern France, and Austria (Riedle-Bauer et al., 2006; Johannesen et al., 2012; Aryan et al., 2014). Several studies have provided evidence that recent *U. dioica-*associated epidemics are related to shifts in *H. obsoletus* populations. These shifts have been attributed to immigration, local demographic expansion, and the evolution of the sympatric host race of *H. obsoletus*. In several cases, the respective expanding vector populations were also responsible for the spread of previously unknown types of “*Ca*. P. solani” (Johannesen et al., 2008; Imo et al., 2013; Johannesen and Riedle-Bauer, 2014). This was seen in Austria for a phytoplasma type named CPs_At1, with the tuf-b2/S6/stamp6/V18 haplotype, which was responsible for an unprecedented *U. dioica*-associated epidemic of BN. At the time of its discovery in 2011, CPs_At1 was already widely distributed in Austria (Aryan et al., 2014).

The study presented here focuses on various aspects of BN epidemiology in Slovenia and Austria. One objective was to reanalyze grapevine samples, for which enough DNA of suitable quality was still available from an official survey of BN in the three wine-growing regions in northeast, southeast, and southwest Slovenia from 2003 to 2016. These samples were used to estimate the molecular diversity of “*Ca*. P. solani” strains using multiple analyses of the *tuf*, *secY*, and *stamp* genes. In addition, the recent situation of the molecular diversity of “*Ca*. P. solani” in grapevines, in reproduction host plants, and *H. obsoletus* in two selected Slovenian wine-growing regions and different regions of neighboring Austria were assessed. We hypothesized that (i) the tuf-b2 type of the *tuf* gene has recently appeared in Slovenian vineyards; (ii) due to the geographical position of Slovenia at the Italian–Slovenian Karst divide, the molecular diversity of “*Ca*. P. solani” differs in southwest Slovenia from that in the northeast and southeast Slovenia; and (iii) the phytoplasma diversity in Austrian Styria is closely related to that of Slovenian Styria.

## Materials and Methods

### Samples, DNA Extraction, and Phytoplasma Testing

As part of the official survey for grapevine yellows diseases carried out by the Administration of the Republic of Slovenia for Food Safety, Veterinary Sector, and Plant Protection, 3,576 symptomatic grapevine samples were collected in different wine-growing regions in Slovenia from 2003 to 2016, and 130 pooled samples that tested positive for “*Ca*. P. solani” were additionally characterized, along with some that tested positive for “*Ca*. P. solani”, *U. dioica*, and *H. obsoletus* samples from 2016 (Table 1). Austrian material (i.e., 357 samples positive for “*Ca*. P. solani” from the grapevine, *U. dioica, C. arvensis*, and *H. obsoletus*) was collected from 2012 to 2019 in Burgenland, Lower Austria, and Styria (Table 1). DNA was extracted from the leaf veins or insects using a cetrimonium bromide extraction procedure modified from Ahrens and Seemuller (1992) or using kits (QuickPick Plant DNA kits; Bio-Nobile, Finland) and a purification system (KingFisher mL; Thermo Scientific, USA), as described by Mehle et al. (2013).

**Table 1:** The total number of samples included in genotyping by sequencing for different “*Ca*. P. solani” hosts.

**Wine-growing region**	**Grapevine**	***Urtica dioica***	***Convolvulus arvensis***	***Hyalesthes obsoletus***
Slovenia NE (Slovenian Styria)	50^*^	2		3
Slovenia SE	32^*^	1		
Slovenia SW	44^*^			
Austria Burgenland	64	2	7	68
Austria Lower Austria	105	4	17	21
Austrian Styria	24	7		38

* *Pooled samples*.

Samples were tested using real-time PCR assays (Abi Prism 7900HT fast detection system; Applied Biosystems) with stolbur (16SrXII) group-specific primers and probe (Hren et al., 2007), as described by Mehle et al. (2013). The final reaction volume of 10 μl contained 2 μl of sample DNA, 1 × TaqMan Universal PCR master mix (Applied Biosystems), 900 nM primers, plus a 250-nM probe. All of the relevant quality controls for PCR-based diagnostics described by Dermastia et al. (2017) were included. A sample was considered positive if it produced an exponential amplification curve that differed from the negative controls. Conversely, if no exponential amplification curve was generated, a sample was considered negative. The 16SrXII-positive samples were further analyzed as described below.

### Molecular Characterization of 16SrXII Isolates

Nested PCR procedures were performed with the following primers: fTUF1/rTUF1 and fTUFAY/rTUFAY (*tuf*; Schneider et al., 1997; POSecF1/POSecR1 and POSecF3/POSecR3 (*secY*; Fialová et al., 2009); StampF/StampR0 and StampF1/StampR1 (*stamp*; Fabre et al., 2011); and StolH10F1/StolH10R1 and TYPH10F/TYPH10R (*vmp1*; Fialová et al., 2009). *Tuf*-, *secY*- and *stamp*-specific PCR assays were performed on a PCR cycler (PCR System 9700 Gene Amp) in 50 μl final reaction volumes that contained 2 μl 10-fold diluted DNA sample, 1 × high fidelity buffer (Invitrogen), 2 mM MgSO_4_ (Invitrogen), 200 μM dNTPs (Applied Biosystems), 0.02 U/μL platinum Taq DNA polymerase (high fidelity; Invitrogen), and 0.2 μM (*sec*Y, *stamp*) or 0.4 μM (*tuf*) of each primer. PCR assays for amplification of *vmp1* were performed on the same PCR cycler, but in 25 μl final reaction volumes that contained 1 μl undiluted DNA sample, 1 × GoTaq Flexi buffer (Promega), 1.5 mM MgCl_2_ (Promega), 200 μM dNTPs (Applied Biosystems), 0.6 U GoTaq DNA polymerase (Promega), and 0.4 μM of each primer. The PCR conditions for the initial denaturation were 2 min (*sec*Y, *stamp*)/3 min (*tuf*)/4 min (*vmp1*) at 94°C, which was followed by 35 cycles of denaturation for 15 s (*sec*Y, *stamp*)/30 s (*tuf*, *vmp1*) at 94°C, annealing for 30 s at 45°C (*tuf*)/52°C (*vmp1*)/54°C (*sec*Y)/56°C (*stamp*), and extension for 1 min at 68°C (*tuf*, *sec*Y, *stamp*)/2 min at 72°C (*vmp1*). The last extension was for 7 min at 68°C (*tuf*)/72°C (*vmp1*). Nested PCR was carried out using 2 μl of the undiluted (*tuf*, *sec*Y, *stamp*) or 30-fold diluted (*vmp1*) PCR amplification product. The nested PCR conditions were as for PCR, except for the annealing temperature (*stamp*, 52°C; *tuf*, 53°C; *vmp1*, 55°C; *sec*Y, 62°C). The nested PCR products were separated on 1% agarose gels that were stained with ethidium bromide and viewed under UV light, with later purification (MiniElute PCR purification kits; Qiagen).

Forward and reverse sequencing reactions for the nested PCR products were performed by Macrogen Europe and LGC (Berlin, Germany), using the Sanger method. The partially amplified sequences of *tuf*, *sec*Y, and *stamp* were compared with sequences from the GenBank database using the BLAST algorithms (http://www.ncbi.nlm.nih.gov/blast). DNA sequence alignments of the Slovenian isolates were carried out by creating contiguities using the ContigExpress software (Vector NTI), and the Austrian isolates were compared in bioedit. (https://bioedit.software.informer.com/).

**Figure 1:**
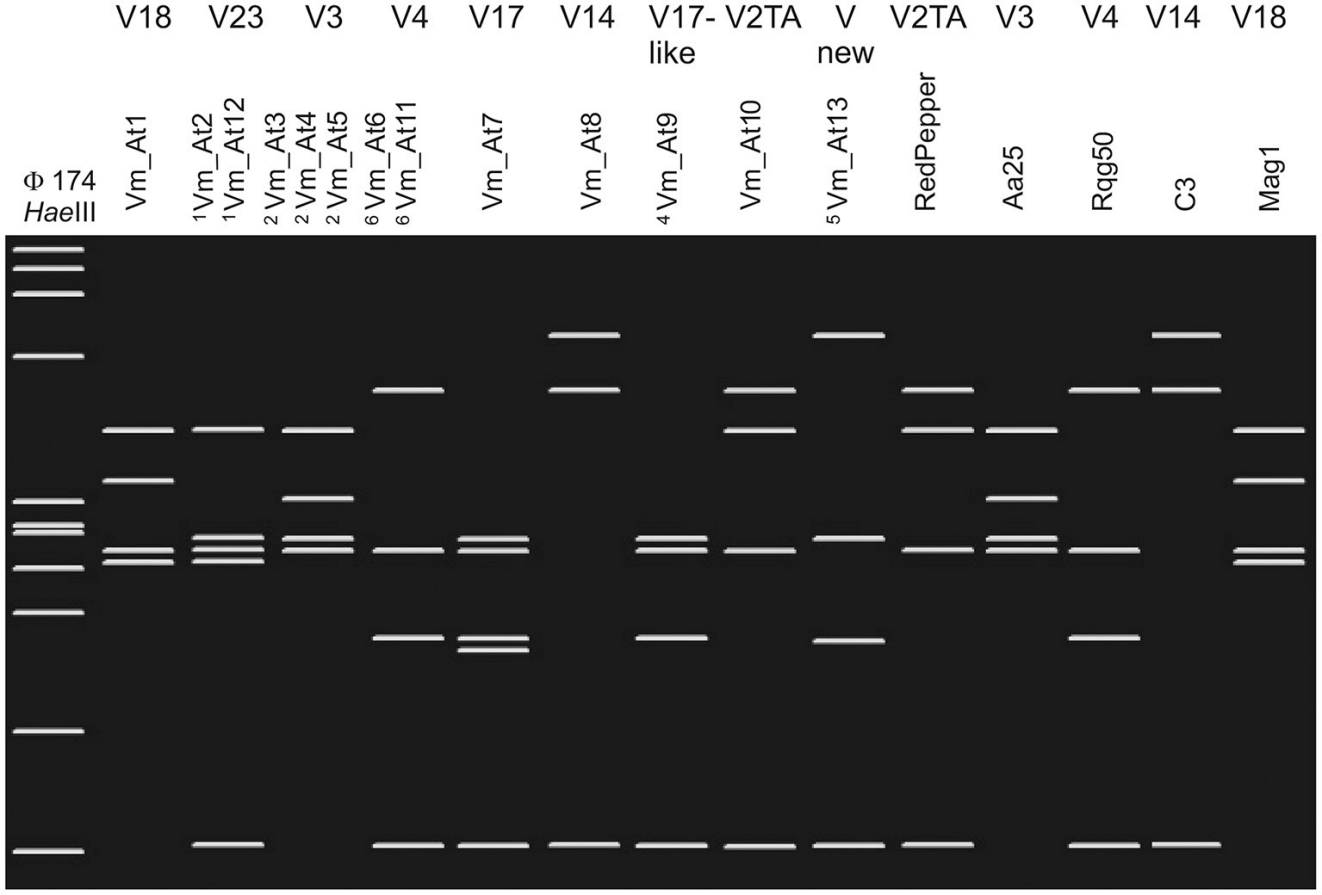
Restriction fragment length polymorphism (RFLP) profiles were obtained after virtual *Rsa*I digestion of trimmed TYPH10F/TYPH10R PCR fragments of *vmp1* of strains collected in vineyard environments in Austria and Slovenia (genotypes Vm_At1–Vm_At13) and the reference strains RedPepper, Aa25, Rqg50, C3, and Mag1. The patterns were generated by pDRAW32 1.1.147. The *Rsa*I-RFLP profiles are labeled on top with the V nomenclature as suggested by the SEE-ERA.NET nomenclature (Foissac et al., 2013). ^1^Genotypes Vm_At2 and Vm_At12 show identical *Rsa*I-RFLP profiles. ^2^Genotypes Vm_At3, Vm_At4, and Vm_At5 show identical *Rsa*I-RFLP profiles. ^3^Genotypes Vm_At6 and Vm_At11 show identical *Rsa*I-RFLP profiles. ^4^The *Rsa*I-profile of Vm_At9 has not been labeled in the V nomenclature, but the pattern is similar to the related V17 profile. ^5^Virtual *Rsa*I digestion results in a novel profile. As marker *Hae*III digested φX174 is shown on the left. The Fragment length from top to bottom are 1,353, 1,078, 872, 603, 310, 281, 271, 234, 194, 118, and 72 bp.

In addition, TYPH10 fragments were analyzed by restriction fragment length polymorphism (RFLP) with *Rsa*I (New England Biolabs) and some TUFAY fragments using *Hpa*II (New England Biolabs), according to the manufacturer instructions. The *Rsa*I and *Hpa*II restriction products were separated by electrophoresis on 2.5% (w/v) polyacrylamide gels, stained with ethidium bromide, and visualized with a UV transilluminator. RFLP patterns of TYPH10 were assigned according to the SEE-ERA.NET nomenclature (Foissac et al., 2013), while RFLP patterns of TUFAY were compared with RFLP patterns identified for “*Ca*. P. solani” by Aryan et al. (2014). Virtual restriction fragment length polymorphism (RFLP) profiles were obtained after *Rsa*I digestion of trimmed TYPH10F/TYPH10R PCR fragments of *vmp1* of strains collected in vineyard environments in Austria and Slovenia (genotypes Vm_At1–Vm_At13) and the reference strains RedPepper (AM992103), Aa25 (HM008614), Rqg50 (KC703033), C3 (HM008603), and Mag1 (HM008613). The virtual gel patterns were generated by pDRAW32 1.1.147 (Figure 1).

## Results

### Diversity of Genotypes in Slovenia and Austria

#### Diversity of the tuf Genotypes

The RFLP patterns of TUFAY fragments of the *tuf* gene identified in the grapevine samples from all Slovenian wine-growing regions (Table 2) were identical to the RFLP patterns previously reported for “*Ca*. P. solani” by Aryan et al. (2014). In the vineyards in the northeast and southeast of Slovenia, the tuf-b restriction profile was confirmed in 81 and 84% of the samples, respectively, whereas in the vineyards in the southwest of Slovenia, the tuf-b was detected in only half of the samples analyzed.

**Table 2:** Restriction fragment (RFLP) patterns of TUFAY of samples from all Slovenian wine-growing regions.

**Wine-growing region**		**2003**	**2007**	**2010**	**2013**	**2015**	**2016**
**Number of samples**							
Slovenia NE (Slovenian Styria)	tuf-a	3	4	2	3	2	0
	tuf-b	3	7	7	29	10	4
Slovenia SE	tuf-a	*/*	2	*/*	6	*/*	*/*
	tuf-b	*/*	15	*/*	28	*/*	*/*
Slovenia SW	tuf-a	7	9	10	9	15	6
	tuf-b	1	30	9	13	8	0

Sequencing of TUFAY amplicons obtained after nested PCR analysis of 65 ‘*Ca*. P. solanis' positive samples from all Slovenian wine-growing regions with tuf-b RFLP patterns revealed two *tuf* sequence types: tuf-b1, which was identical to the Austrian sequence KJ469708; and tuf-b2, which was found for the first time in Austria (Accession No. KJ469709) associated with the *U. dioica's* wild reservoir in September 2011 (Aryan et al., 2014) (Supplementary Tables 1–3).

An interesting observation was the tuf-b2 genotype in 50% of the samples of grapevines from 2003 from different vineyard sites in Slovenian Styria, in the northeast of Slovenia, near the border with Austria (Supplementary Tables 1–3). The other half of the isolates tested had the tuf-a genotype. For the grapevine samples from 2007, all three of the genotypes of the *tuf* gene (i.e., tuf-a, tuf-b1, and tuf-b2) were detected in approximately equal proportions. However, in the following years, the proportion of tuf-b2 increased. Therefore, in 2015, the tuf-b2 proportion was 75%, while tuf-b1 was detected in 8.3% and tuf-a in 16.7%. In 2016, four additional samples of grapevines and one sample of *U. dioica* were collected in Slovenian Styria, and the tuf-b2 genotype was confirmed for all these samples. *H. obsoletus* collected in Slovenian Styria in 2016 tested positive for “*Ca*. P. solani” in four cases, and tuf-b2 was confirmed for three of them (Supplementary Tables 1–3).

Although the RFLP analysis with *Hpa*II of the grapevine samples from southeast Slovenia (near the border with Croatia) from 2007 and 2013 revealed a high proportion of samples with a tuf-b restriction profile (Table 2), in 2016, a tuf-a sequence was detected for all six grapevine samples from the southeastern part of Slovenia (Supplementary Tables 1–3). However, at the same site, tuf-b2 was confirmed in one sample of *U. dioica* (Supplementary Tables 1–3). Several *C. arvensis* plants collected at the same sampling site in 2016 were not infected with phytoplasma.

Sequence analysis of grapevine samples with a tuf-b restriction profile from southwest Slovenia on the border with Italy (Table 2) revealed the presence of tuf-b1 only (Supplementary Tables 1–3). This genotype represented about a half and the second half was tuf-a (Table 2).

In Austria, the first characterization of tuf types for grapevines, *U. dioica*, and *C. arvensis* collected in Burgenland, Lower Austria, and Austrian Styria was carried out from 2005 to 2007. The tuf-b restriction profile only was detected for all of these samples, including for *U. dioica* from three different locations in Austrian Styria. A more extensive characterization of the phytoplasma types involved, however, was not carried out at that time (Riedle-Bauer et al., 2008). Unfortunately, the DNA of these samples was not available anymore. More in-depth information on the BN types in Austria was collected in a subsequent analysis from 2012 to 2019, which again included samples of grapevines, *H. obsoletus, U. dioica*, and *C. arvensis* from the same area (Supplementary Tables 4–6). Earlier observations in 2012 and 2013 had shown the occasional occurrence of the tuf-a profile in one site in Burgenland (<5% in Rust) and Styria (<17% in Kitzeck), respectively, for these *H. obsoletus* samples (Supplementary Tables 4–6; Aryan et al., 2014). Later, in 2017, the tuf-a profile was detected only for one grapevine sample in Burgenland and one *H. obsoletus* sample in Styria, and in 2019, the tuf-a profile was detected for two *H. obsoletus* samples in Styria (Kitzeck), which accounted for 29% of all of the BN-positive *H. obsoletus*; in contrast, in 2018, the tuf-a genotype was not detected for this area. The tuf-b1 genotype prevailed for grapevine samples in Lower Austria in the area of Falkenstein in 2012, 2017, and 2018, and in Burgenland in Horitschon and Zagersdorf in 2017 and 2018, whereas the tuf-b2 type prevailed for all of the other locations and all of the other years. While samples of *H. obsoletus* contained mainly tuf-b2 for all the wine-growing regions and originated from *U. dioica*, the situation was different in 2018 at a site in Lower Austria and in 2019 in Lower Austria and Styria, with increasing numbers of tuf-b1 that were associated with *C. arvensis*. Only tuf-b2 was detected for *U. dioica* and only tuf-b1 for *C. arvensis*.

#### Diversity of the secY Genotypes

Phylogenetic analysis of the *sec*Y gene for grapevine samples from Slovenia and Austria showed the affiliation with six secY groups according to the SEE-ERA.NET nomenclature (Foissac et al., 2013), with a clear geographical distribution (Supplementary Tables 1–6). For easier comparison with data from the scientific literature, which uses different nomenclature, see Supplementary Table 7.

Five *secY* genotypes were detected over the years in southwest Slovenia at the border with Italy, all of which had already been detected in other countries for different insect or plant hosts. Among these, only the secY-1 genotype was associated with tuf-b1 for all the samples and it occurred in 35.9% of samples. SecY-41 was detected in several samples and was associated with a tuf-a type in most cases (Supplementary Tables 1–3). However, in almost 29% of the samples, secY-41 was associated with tuf-b1. Another genotype that occurred for grapevines from southwest Slovenia and was associated with tuf-a was secY-6. However, it was also detected in a quarter of the samples with the tuf-b1 genotype. A similar distribution between tuf-a and tuf-b1 was shown for the secY-7 genotype (Supplementary Tables 1–3). On the contrary, the secY-4 genotype prevailed in haplotypes with tuf-b1 but was also detected in a sample with tuf-a (Supplementary Tables 1–3).

In the northeastern part of Slovenia bordering Austria, only the secY-6 and secY-41 genotypes, and in a single case the secY-4 genotype, were detected (Supplementary Tables 1–3). SecY-6 prevailed, and its proportion increased in the years from 2003 to 2015, from 50 to 91.7%, respectively. In 2016, the secY-6 genotype was detected for four out of five samples (Supplementary Tables 1–3). Moreover, this secY genotype was associated with a tuf-b2 type in 77% of the samples in northeast Slovenia (Supplementary Tables 1–3), which was the case for all the Austrian samples regardless of the sample source (grapevines or *H. obsoletus*) (Supplementary Tables 4–6). However, in 2013 and 2015, the tuf-a/secY6 combination was also detected (Supplementary Tables 1–3). A prevalence of 67% of the secY-6 genotype was also confirmed in 2016 in the southeastern part of Slovenia on the border with Croatia (Supplementary Tables 1–3). Interestingly, this occurred for four out of five grapevine samples associated with tuf-a. Similar to southwest Slovenia, secY-41 was detected in association with tuf-a in northeast Slovenia from 2003 to 2015.

In Austrian samples (Supplementary Tables 4–6), the vast majority of sec types were secY-1 and secY-6, with secY-1 associated with tuf b1 and secY-6 associated with tuf-a or tuf-b2. SecY-4 was only found in Lower Austria for a single grapevine sample and four *H. obsoletus* samples between 2012 and 2019 and was always associated with tuf-b1. SecY-7 and secY-41 were found in different years for three and four *H. obsoletus* samples, respectively, in Styria only, and secY-7 was found for a single grapevine sample in Burgenland; all here were detected in association with tuf-a. In Lower Austria, a novel secY-type was seen for two grapevine samples, which was closely related to secY-6 (1 mismatch) and was associated with tuf-b2. In all the Austrian samples, there was a strict association of tuf-b1 with secY-1 and secY-4, and of tuf-a and tuf-b2 with secY-6, secY-6-like, secY-7, and secY-41.

#### Diversity of the Stamp Genotypes

Phylogenetic analysis of *stamp* genotypes revealed 13 different genotypes, named according to the SEE-ERA.NET nomenclature (Foissac et al., 2013). For easier comparison with data from the scientific literature, which uses a different nomenclature, see Supplementary Table 7. (Supplementary Tables 1–6). Again, their geographical distribution was very clear.

Of the nine different *stamp* genotypes detected in these Slovenian wine-growing regions, all except stamp-6 were detected in southwest Slovenia near the border with Italy (Supplementary Tables 1–3). The stamp-46 genotype was present in all sampling years and for most of the samples associated with tuf-a (Supplementary Tables 1–3). The sequence corresponding to the stamp-19 genotype was detected in 21% of the isolates and the stamp-22 genotype was seen in 18% of the isolates (Supplementary Tables 1–3). In addition, the stamp-59 genotype occurred here three times in association with tuf-a and once with tuf-b1.

In marked contrast to the southwest wine-growing region of Slovenia, 72% of the samples in northeast Slovenia were associated with the stamp-6 genotype in combination with either tuf-b1 or tuf-b2, but never with tuf-a (Supplementary Tables 1–3). In 2016, all the isolates from grapevines in northeast Slovenia had the stamp-6 genotype. The stamp-6 genotype was also detected for *U. dioica* and *H. obsoletus* samples from northeast Slovenia in 2016 (Supplementary Tables 1–3). The stamp-6 genotype was also prevalent in southeast Slovenia in 2016 (Supplementary Tables 1–3). In addition, stamp-46 was detected in 21% of the isolates from grapevines in northeast Slovenia. It is of note that stamp-46, which is associated with tuf-a, accounted for half of all the samples from northeast Slovenia in 2003, while its proportion decreased over the following years, such that it was found for only one isolate in 2015. In addition to stamp-6 and stamp-46, the genotype stamp-29 was detected for one grapevine sample in 2013 (Supplementary Tables 1–3). The stamp-59 genotype occurred here three times in association with tuf-a and once with tuf-b1 (Supplementary Tables 1–3).

The variability of the *stamp* gene was higher in the Austrian wine-growing regions. In addition to the clearly predominant genotypes of stamp-6 and stamp-9, the following genotypes were detected: stamp9D, stamp-22, stamp-23, stamp-4, stamp-46, stamp-19, stamp-29, stamp-50, and stamp-52. Furthermore, a novel stamp-52-like genotype was detected for two grapevine samples from Lower Austria. The same combination of tuf-a and stamp-46 as in Slovenia was detected for four *H. obsoletus* samples from Austrian Styria in 2013 (Supplementary Tables 4–6).

### Haplotypes

**Figure 2:**
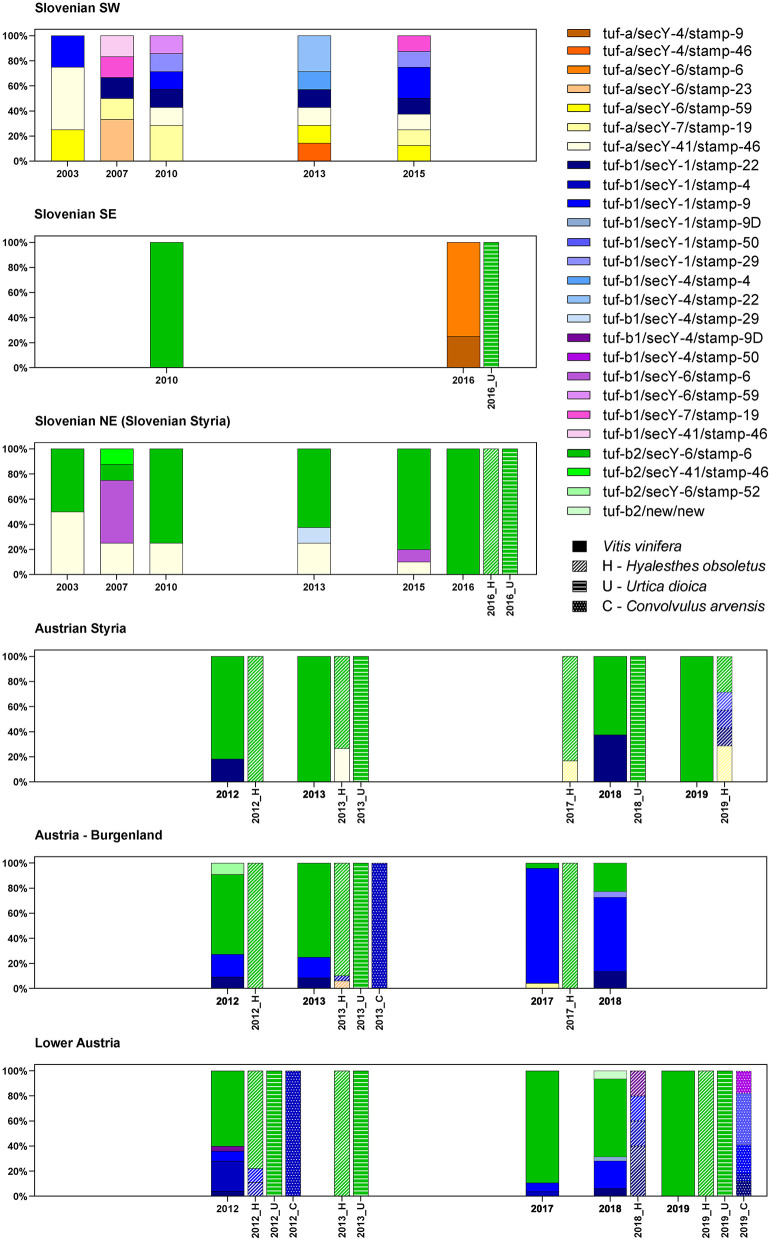
Spatial and temporal diversity of the haplotypes associated with “*Ca*. P. solani” in Slovenia and Austria.

Twenty-four different haplotypes were recognized for Slovenia and Austria, with the highest diversity in southwest Slovenia, bordering Italy (Figure 2).

#### High Haplotype Diversity at the Border With Italy

In southwest Slovenia, the grapevine samples allowed detection of 13 different “*Ca*. P. solani” haplotypes, with no apparent temporal distribution (Figure 2). Across all the sampling years, there were similar distributions of haplotypes associated with the *U. dioica* (tuf-a) and *C. arvensis* (tuf-b) cycles. There were fewer haplotypes detected in 2003, but this appeared to be due to fewer samples being analyzed (Figure 2). However, two of four samples in 2003 harbored the *U. dioica* associated haplotype tuf-a/secY-41/stamp-46.

#### Prevalence of the tuf-b2/S6/stamp-6 Haplotype in Slovenian Styria and Austria

In contrast to southwest Slovenia, only five tuf/stamp/secY “*Ca*. P. solani” multilocus haplotypes were detected in Slovenian Styria from 2003 to 2016 (Figure 2). However, the tuf-a/secY-41/stamp-46 haplotype was the only one detected in both Slovenian Styria and southwest Slovenia. Moreover, it occurred in the same years. The proportion of the tuf-a/secY-41/stamp-46 haplotype decreased in Styria over the years and was only 10% in 2015, while it was not detected in 2016 (Figure 2). This haplotype has been designated as CPsM4_At4 (Aryan et al., 2014), and in 2013, it was also reported occasionally for *H. obsoletus* in Austria (Figure 2).

In two individual samples (one from 2007, one from 2013), the tuf-b1/secY-4/stamp-29 and tuf-b2/secY-41/stamp-46 haplotypes were detected (Figure 2). Neither of these haplotypes has been reported previously. Of note, the tuf-b1/secY-6/stamp-6 haplotype was also detected (Figure 2, Supplementary Tables 1–3), where the secY-6 genotype has been associated with *U. dioica*, while tuf-b1 is characteristic of the *C. arvensis* cycle. However, as most of the archived Slovenian samples were collected during official state surveys, in many cases, symptomatic samples were pooled. Therefore, in at least two samples (as indicated in Supplementary Tables 1–3), mixed infection with tuf-b2 cannot be excluded. This tuf-b1/secY-6/stamp-6 haplotype has never been reported for Austria previously.

In Slovenia, the tuf-b2/secY-6/stamp-6 haplotype was detected across all the years studied here, and it was predominant in 2010, 2013, and 2015, while also being the only haplotype detected for grapevines, as well as for *H. obsoletus* and *U. dioica* in 2016 (Figure 2, Supplementary Tables 1–3).

The proportion of the tuf-b2/secY-6/stamp-6 haplotype remained high for grapevine samples from Austrian Styria and Lower Austria, where it was the only haplotype detected in 2019. In 2017, it was the only haplotype detected in *U. dioica*, and it was also present in a high proportion of *H. obsoletus* samples taken across all these wine-growing regions. It also remained the dominant genotype in *H. obsoletus* in 2019 (Figure 2, Supplementary Tables 4–6). In 2017, a tuf-a/secY-7/stamp-19 haplotype appeared for the first time for *H. obsoletus* samples from Austrian Styria, and its proportion increased to 29% in 2019. In Lower Austria, four haplotypes were detected for *H. obsoletus* samples in 2018, as tuf-b1/secY-1/stamp-22, tuf-tuf-b1/secY-1/stamp-4, tuf-b1/secY-1/stamp-9, and its variant tuf-b1/secY-1/stamp-9D. In contrast, in 2019, the only haplotype detected for *H. obsoletus* samples in Lower Austria was the predominant tuf-b2/secY-6/stamp-6 haplotype, which did not occur for a single *C. arvensis* sample from the same site, and so appears to be of *U. dioica* origin (Figure 2, Supplementary Tables 4–6). In Burgenland, the proportion of the tuf-b2/secY-6/stamp-6 haplotype decreased from 2013 to 2018, and the most important haplotype detected was tuf-b1/secY-1/stamp-9 (Figure 2, Supplementary Tables 4–6). However, it should be noted that between the years, the sample sites were not the same, so it is unclear whether the observed changes in haplotypes are a reflection of possible shifts toward new epidemic cycles in the coming years, or if these are observations of more stable regional variations.

#### The Southeastern Border With Croatia Is Characterized by Northern and Unknown Haplotypes

Multilocus genotyping of samples from southeast Slovenia confirmed the *U. dioica*-associated haplotype tuf-b2/secY-6/stamp-6 for one grapevine sample from 2010 (Supplementary Tables 1–3), and one *U. dioica* sample from 2016 (Figure 2, Supplementary Tables 1–3). Interestingly, the 2016 grapevine samples from the same site in southeast Slovenia showed haplotypes that consisted only of the *U. dioica*-associated tuf-a genotype (Figure 2, Supplementary Tables 1–3).

## Discussion

### Diversity of Genotypes and Haplotypes

The spatial diversity of genotypes associated with “*Ca*. P. solani” in Slovenia and Austria was high in southwest Slovenia on the border with Italy, and moderate in the other wine-growing regions studied (Figure 2, Supplementary Tables 1–4). In addition, temporal changes in genotype diversity over 16 years were moderate in all of these regions (Figure 2, Supplementary Tables 1–4) (Aryan et al., 2014). All the genotypes revealed for the *tuf*, *secY*, and *stamp* genes have been reported previously. The only exceptions were one *secY* and one *stamp* genotype from the same two grapevine samples in Lower Austria (Supplementary Tables 4–6). On the other hand, few new sequences for the *vmp1* gene were detected in Austria (Figure 1; Supplementary Tables 4–6). However, the RFLP profile V3 or sequences that matched this profile were common in all wine-growing regions in Slovenia and Austria (Figure 1, Supplementary Tables 1–6). Considering the haplotypes revealed, some previously unreported combinations of the *tuf*, *secY*, and *stamp* genotypes were detected (Figure 2, Supplementary Tables 1–3). Of note, as indicated in Supplementary Tables 1–3, some combinations had been previously reported as specifically associated with *U. dioica* tuf-a/b2 or *C. arvensis* tuf-b1, but some deviations from the rule were detected here.

The analysis of the *tuf* gene in grapevine plants infected with “*Ca*. P. solani” from southwest Slovenia on the border with Italy (Table 2) mirrors the situation in central Italy, which also confirmed only the presence of tuf-a and tuf-b1 (Landi et al., 2019). The discovery of tuf-b2 in Austrian Styria in 2011 (Aryan et al., 2014) and its confirmation in grapevines and *H. obsoletus* samples in Croatia (Plavec et al., 2018) and Montenegro (Kosovac et al., 2016) from 2009 to 2017 could be related to its sudden appearance at that time. On the other hand, it could also be a result of the new methodological approaches involving sequencing that allowed determination of tuf-b2, as RFLP analysis of TUFAY fragments with *Hpa*II does not allow discrimination between tuf-b1 and tuf-b2. Although tuf-b2 shows a tuf-b RFLP profile, its sequence is intermediate between tuf-a and tuf-b1 (Aryan et al., 2014). However, analysis of samples collected in Slovenian Styria in 2003 and re-examined as part of this study and also the tuf-b types that were recorded for *U. dioica* samples in Austrian Styria already from 2005 to 2007 (Riedle-Bauer et al., 2008), support this methodological explanation, at least for some of the samples collected from 2003 onward.

Among *secY* genotypes detected, only the secY-1 was associated with tuf-b1 for all of the samples, which is in agreement with the presumed association with the *C. arvensis* cycle (Johannesen et al., 2012). This secY-1 genotype is widely distributed and has been recorded for grapevines in Serbia (Accession No. JX645768), for *H. obsoletus* in Austria (Aryan et al., 2014) and France (Fabre et al., 2011), for *Salvia sclarea* in France (Accession No. LT841330.1), for *Solanum lycopersicum* in France (Accession Nos. AM992086 and KT310185) and Azerbaijan (Accession No. LT899858), and *Repatalus panzeri* in Serbia (Accession No. KC703048) and Switzerland (Accession No. KP635228). The secY-41 genotype was previously detected in southwest Slovenia for *H. obsoletus* associated with *U. dioica* (Johannesen et al., 2012). Another genotype presumably associated with *U. dioica* that occurred in grapevines from southwest Slovenia was secY-6 (Johannesen et al., 2012). Of note, in some Slovenian grapevine samples, both genotypes were also detected in several samples with the tuf-b1 genotype, which were presumably associated with *C. arvensis* (Johannesen et al., 2012) but are different from tuf-b2 by only a single mutation. The detected secY-7 genotype was previously reported for grapevines in Italy (Trivellone et al., 2016) and Croatia (Accession No. HQ413162) and for *H. obsoletus* in Austria (Aryan et al., 2014). The secY-4 genotype has been shown for grapevines in Austria and Croatia (Aryan et al., 2014; Plavec et al., 2015) and for *C. arvensis* in Croatia (Plavec et al., 2015). The secY-6 genotype, a dominant secY genotype in northeast Slovenia and Austria, was previously reported for grapevine and *H. obsoletus* associated with “*Ca*. P. solani” in Austria (Aryan et al., 2014), Italy (Murolo and Romanazzi, 2015), and Croatia (Plavec et al., 2015).

The prevalence of the stamp-6 genotype is consistent with data from Austria and Croatia, where the stamp-6 genotype was a major *stamp* genotype for grapevines and *H. obsoletus* captured on grapevines, and for *U. dioica* (Aryan et al., 2014; Plavec et al., 2015, 2018). The stamp-46 genotype found in grapevine in southeast Slovenia had previously been reported for *U. dioica* from the same area (Johannesen et al., 2012). The stamp-19 genotype has been reported for grapevines from Croatia (Accession No. FN813266) (Fabre et al., 2011) and Italy (Accession No. MW759853) (Contaldo et al., 2021), while stamp-22 is known to be associated with stolbur disease in potato in Serbia (Accession No. KP877596) (Mitrović et al., 2016), pepper in Bosnia and Herzegovina (Accession No. KU295501) (Delić et al., 2016) and was also found for an isolate of the carrot *Daucus carota* in the southeastern part of Slovenia (Mehle et al., 2018). *Stamp* genotypes have been mainly recorded for isolates from different plant hosts and insects from Serbia (Cvrković et al., 2014; Mitrović et al., 2016; Kosovac et al., 2019; Contaldo et al., 2021). The *stamp* sequence of the stamp-4 genotype was discovered for the periwinkle *Catharanthus roseus* experimentally infected by *H. obsoletus*, the leafhopper *Anaceratagallia ribauti* in Austria (Aryan et al., 2014), grapevine in Italy, Hungary, and Germany (Fabre et al., 2011; Pierro et al., 2018; Contaldo et al., 2021), tomato in Italy, as well as a potato in Hungary (Contaldo et al., 2021).

Despite the high diversity of haplotypes in southwest Slovenia, they were all associated with “classical” either *U. dioica* (tuf-a) or *C. arvensis* (tuf-b1) cycles in the area. It is noteworthy that individuals of *H. obsoletus* have already been reported from southwest Slovenia that either possessed the genotype tuf-a and were caught on *U. dioica* or tuf-b and were caught on *C. arvensis* (Johannesen et al., 2012). According to the official monitoring of *H. obsoletus* in vineyards in Slovenian Styria in 2002, *H. obsoletus* was widespread in vineyards where *C. arvensis* was abundant in the green cover, although *U. dioica* was also present (Seljak et al., 2003). *H. obsoletus* has been widespread in Slovenia since 1999 (Seljak Gabrijel, personal communication, http://www1.pms-lj.si/animalia/load.php?species=4983) and was mainly associated with *U. dioica*, and to a lesser extent with *C. arvensis*. This is consistent with the predominance of the tuf-b2/secY-6/stamp-6 haplotype, which has also been associated with *H. obsoletus* and *U. dioica* as a source of phytoplasma in plants in Austria (Aryan et al., 2014). The tuf-b2/secY-6/stamp-6 haplotype was previously reported as CPsM4_At1 in Austria and is also a dominant haplotype in neighboring Croatia (Plavec et al., 2018). Its increase in Austria was seen here from 2011 onward. It prevailed for *H. obsoletus* after 2012 in different locations in Austria. Moreover, the tuf-b2/secY-6/stamp-6 haplotype coincided with a drastic change in the ‘*Ca*. P. solanis' epidemiology in Austria after 2012, where there was a sudden increase in the population density of *H. obsoletus*, with its association mainly with *U. dioica* instead of *C. arvensis* (Aryan et al., 2014).

### Then, It Gets Even More Complicated

**Figure 3:**
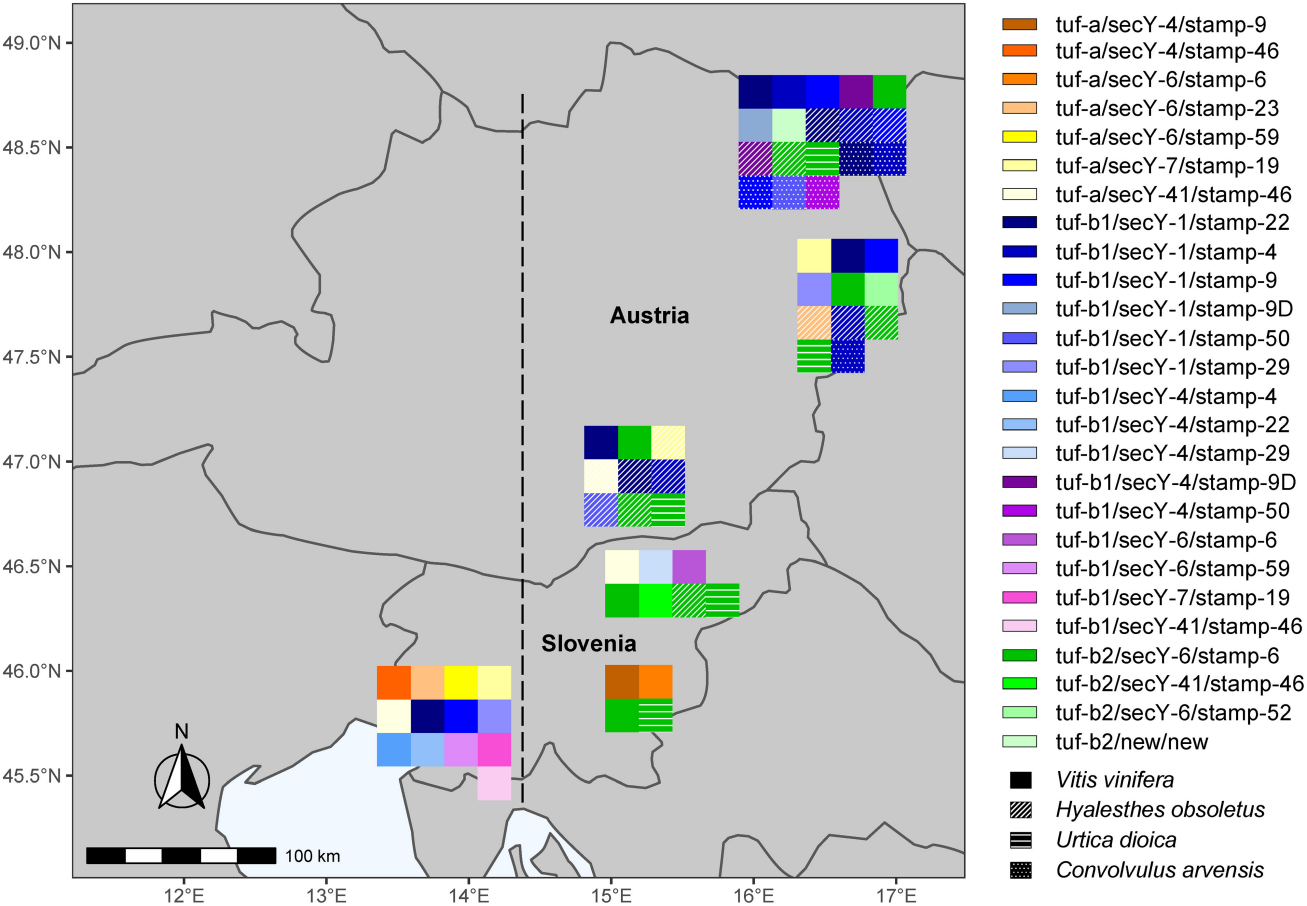
All detected haplotypes of “*Ca*. P. solani” in wine-growing regions in Slovenia and Austria. The line denotes the Italian-Slovenian Karst divide.

The results of the present study confirm our hypothesis that due to the geographical position of Slovenia at the Italian–Slovenian Karst divide, the molecular diversity of “*Ca*. P. solani” west of this geographic line differs from the wine-growing regions in Slovenia and Austria investigated here located east of the Italian–Slovenian Karst divide (Figure 3). Moreover, the striking result is the higher variability of the haplotypes in southwest Slovenia compared to the haplotypes in the more eastern wine-growing regions. However, the second most common “*Ca*. P. solani” haplotype for grapevine samples from northeast Slovenia, tuf-a/secY-41/stamp-46, was also present in southwest Slovenia (Figures 2, 3, Supplementary Tables 1–3). Furthermore, the tuf-a/secY-41/stamp-46 haplotype from southwest Slovenia has also been detected for Austrian *H. obsoletus* samples and was designated as CPsM4_At4 (Aryan et al., 2014), while the tuf-a/secY-6/stamp-59 haplotype has never been detected in Austria. This finding might be explained by a previous study of *H. obsoletus* populations based on microsatellite allele frequencies, in which two Slovenian planthopper populations from southwest Slovenia were related to both, Adriatic and Pannonian populations (Johannesen and Riedle-Bauer, 2014). On the other hand, in the same study, the vast majority of individuals from Burgenland and Lower Austria were assigned to the Pannonian population (Johannesen and Riedle-Bauer, 2014). The absence of the tuf-b2/secY-6/stamp-6 haplotype for grapevine samples from southwest Slovenia and its dominant presence in Austria and northeast Slovenia (this study) and in Croatia (Plavec et al., 2018) might indicate the Pannonian origin of this haplotype (Figure 3). Another interesting comparison between southwest Slovenia and northeast Slovenia shows that several *tuf* and *sec*Y genotypes occurred in both regions, while most *stamp* genotypes occurred only in one region. It has been suggested that Stamp membrane proteins are involved in the interactions of “*Ca*. P. solani” with its hosts (Cimerman et al., 2009; Fabre et al., 2011) and that sequence mutations in their coding genes are related to the geographical distribution and host range of “*Ca*. P. solani” (Murolo et al., 2010, 2014; Johannesen et al., 2012; Cvrković et al., 2014). Despite the small size of Slovenia, the present data support this idea and show that several different epidemiological cycles of “*Ca*. P. solani” are probably present simultaneously, which can be confirmed by further studies of the vectors and hosts of this phytoplasma. How and whether the haplotype changes in *H. obsoletus* and *C. arvensis* associated with “*Ca*. P. solani” are related to new epidemiological cycles of this phytoplasma is difficult to predict, where they might be associated with new insect vectors and new plant sources, and/or with changes in climate and land use. Therefore, the successful implementation of BN disease management based solely on monitoring molecular variants of these phytoplasma types remains extremely complex.

## Data Availability Statement

The datasets presented in this study can be found in online repositories. The names of the repository/repositories and accession number(s) can be found in the article/Supplementary Materials.

## Author Contributions

NM participated in the design of the study, writing of the manuscript, and supervised along with SK, SM, and SV during the performance of the experiments. SK, SM, SV, and AK performed the genotyping analyses. MP collected material and participated in the writing of the manuscript. MR-B and GB collected the material, performed the experiments in Austria, and participated in the writing of the manuscript. MD participated in the design of the study, coordinated the study, and wrote and finalized the manuscript. All authors contributed to the article and approved the submitted version.

## Funding

This study was supported by the Slovenian-Austrian research grant J1-7151 funded by the Slovenian Research Agency and the Austrian Science Fund (FWF), with grants I 2763-B29 and I-5042B.

## Conflict of Interest

The authors declare that the research was conducted in the absence of any commercial or financial relationships that could be construed as a potential conflict of interest.

## Publisher's Note

All claims expressed in this article are solely those of the authors and do not necessarily represent those of their affiliated organizations, or those of the publisher, the editors and the reviewers. Any product that may be evaluated in this article, or claim that may be made by its manufacturer, is not guaranteed or endorsed by the publisher.
